# In Situ High Shear Perturbation of Surface Molecular Layers with Neutron Reflectivity

**DOI:** 10.1021/acs.langmuir.6c03616

**Published:** 2026-08-10

**Authors:** Najib Sharifi, Rebecca J. L. Welbourn, Stuart M. Clarke

**Affiliations:** 1 Institute for Energy and Environmental Flows and Yusuf Hamied Department of Chemistry, 2152 University of Cambridge, Cambridge CB2 1EW, U.K.; 2 ISIS Neutron & Muon Source, Rutherford Appleton Laboratory, Science and Technology Facilities Council, Chilton, Didcot, Oxon OX11 0QX, U.K.; 3 Neutron Scattering Division, Oak Ridge National Laboratory, Oak Ridge, Tennessee 37831, United States

## Abstract

We present the effect of in situ imposed shear on two adsorbed molecular layers using a flow cell that enables access to shear rates of up to 2 × 10^5^ s^–1^ (shear stress: 2 × 10^2^ Pa). The response of these molecular layers to shear is determined by the relative strength of the surface binding and the imposed shear. An adsorbed bilayer of palmitic acid, weakly adsorbed on a solid silicon surface, is found to be readily removed. In contrast an adsorbed bilayer of sodium bis­(2-ethylhexyl) sulfosuccinate is essentially unchanged with the highest shear we could impose, despite being completely removed by rinsing with pure water (i.e., “dissolving” the layer). The surface binding strength of each species is independently captured through adsorption measurements. These results show, by direct in situ measurement, that whether a surface adsorbed additive is perturbed by an imposed high shear field depends on the nature of the adsorbed layer and the strength of its binding to the surface relative to the applied shear stress rather than the magnitude of the shear stress alone. This is an important finding with direct implications for the design and selection of additives in lubricant and anticorrosion formulations, where retention of a protective molecular layer under high-shear boundary conditions governs the performance.

## Graphical abstract

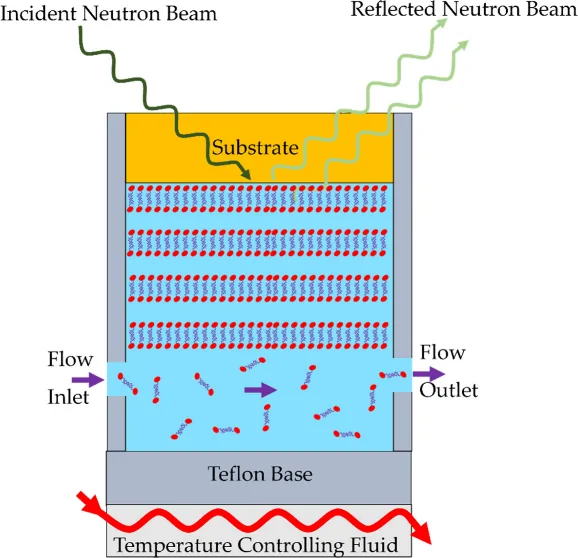

## Introduction

Many commercial systems involve rubbing surfaces and under high loads. This can lead to wear and failure. For example, the increasing size of wind turbines leads to high loads of the order of GPa^1^ in the gearbox which may lead to damage, high repair costs, and downtime.^2,3^ This is especially a challenge where devices are inaccessible offshore. To prevent wear and surface degradation in such systems, a number of approaches are used. Typically, liquid lubricants that act through viscosity are ineffective as they are “squeezed” out at such high loads in this so-called “boundary” regime. Particular hard, wear resistant, “inorganic” surface coatings may be used, such as diamond-like coatings (DLC),^4,5^ but these can be costly to apply. More generally molecular additives, which are proposed to adsorb on the surface of interest from a solvent (for example, as part of a lubricant formulation), can reduce friction and help prevent wear. However, it is not known if high shear fields present at the surface (from flowing liquids or rubbing) remove these molecular layers that would lead to a deterioration in performance. It is experimentally very challenging to directly characterize the very small molecular layers and their changes at the solid/liquid interface under the very high shear fields of interest. This is the central issue of this present work where neutron reflectivity is used to investigate adsorbed surface layers in situ under shear. Neutron reflectivity is particularly well suited to probing such interfacial systems because it provides subnanometer resolution of the structure and composition of thin adsorbed layers at buried solid/liquid interfaces in situ.

Several workers have applied neutron reflectometry to investigate high shear fields at the solid/liquid interface, with a variety of sample environment approaches. A study by Welbourn et al.^6^ used neutron reflectivity (NR) with rheology (cone and plate rheometer) to measure the shear response of an adsorbed anionic surfactant (sodium bis­(2-ethylhexyl) sulfosuccinate, AOT) at the alumina–water interface. They report that at low surfactant concentrations where a single bilayer adsorbs at the interface, the structure of the bilayer adsorbed directly on the solid surface does not change with the imposed shear (oscillatory or steady) up to approximately 500 s^–1^, the maximum accessible with this equipment. These adsorbed AOT bilayers are expected to be very thin, 20–30 Å thick.

However, a higher concentration surfactant solution of AOT forms a stack of bilayers that form a multilayer pile on the surface. These bilayers are essentially aligned parallel to the solid substrate and propagate out into the solution. The interbilayer spacings are 100s Å (with water in the interbilayer regions), and there can be many bilayers in a stack. Hence, overall, these are much thicker adsorbed layers. Under steady shear, the multilayers are greatly affected and, in some cases, the outer bilayers are lost completely. However, even at the highest shear available, the bilayer that is bound to the alumina surface remains intact on the solid surface.

Beyond the cone-and-plate rheo-NR approach, tribometer-coupled reflectometry has also been developed. Armstrong et al.^7^ reported a neutron and X-ray reflectometry sample environment using an entrainment/tribometer geometry, in which glycerol monooleate adsorbed at an iron oxide–dodecane interface gave an interfacial layer of approximately 24–26 Å that appeared largely unchanged at a shear rate of 7 × 10^2^ s^–1^. This was an important proof of principle for reflectometry during rubbing/entrainment, although the accessible shear rates remained modest. More recently, Shaffer et al.^8^ introduced a linear reciprocating tribometer for in situ NR with uniform velocity over a large illuminated region and independent control of normal force and sliding velocity, though their proof-of-principle measurements focused on low-force soft-matter contacts rather than high-load boundary-lubrication conditions. Another method that can access higher shear rates at the solid–liquid interface for neutron reflectivity studies is use of a flow cell as successfully employed by Penfold et al.^9^ The flow cell produces laminar flow (Poiseuille shear) between 2 parallel plates resulting in a high shear adjacent to the solid surface of interest. Penfold et al. employed this cell to characterize a multilayer of nonionic surfactant at the silicon–water interface, which became more ordered on application of a shear gradient above 10^4^ s^–1^. Another study employing a similar cell to study polyelectrolyte multilayers reported a structural change resulting in the degree of solvation decreasing on shear while the layer thickness remain the same.^10^ In a more recent study employing a similar narrow gap flow cell, with increased shear rate of 10^5^ s^–1^, Ivkov et al.^11^ demonstrated that long chain polystyrene (80 000 MM) polymers chemically tethered to a silicon surface remains unchanged even at the highest shear.

Yamashita et al.^12^ combined neutron reflectometry with a parallel-face narrow-gap viscometer designed to maintain a constant micrometre-scale gap under lubrication-relevant conditions. Using a 2.3 μm gap and a shear rate of 6 × 10^4^ s^–1^, they showed that an adsorbed polymethacrylate layer on Cu became both thinner and less dense under shear, with approximately 74% of the adsorbed polymer removed. This study is particularly relevant because it demonstrates that once smaller gaps and higher shear fields are accessed, in situ NR can directly detect shear-induced depletion of an adsorbed layer.

Complementary insight has come from molecular simulation, notably the MD and nonequilibrium MD studies of Camp and co-workers^13^ on fatty-acid, amine, and glycerol monooleate films under confinement and shear, which have clarified how adsorption, molecular tilt, and self-assembly influence frictional response. However, the simulated shear rates (10^8^–10^10^ s^–1^) are far above those accessible experimentally, so these studies are best viewed as mechanistic rather than directly comparable.^13,14^

It has been reported in the literature that corrosion rates increase under shear, which has been attributed to a protective organic layer being “knocked off” due to shear induced by the fluid flow.^15,16^ However, this corrosion study was not able to access the adsorbed layer structure directly, and it is not known whether other effects of the flow (such as better mass transport of corroding species) may be responsible rather than removal/perturbation of the adsorbed surfactant layer.

The effect of shear on adsorbed molecular species has also been considered in mechanochemistry where the mechanical shear force couples with thermal energy to help facilitate bond cleavage and reduce reaction energy barriers (for desorption/reaction).^17^ There is a very significant body of important work involving tribometer friction tests characterizing the surface-adsorbed layers.^18–22^ However, in this mechanochemistry study, two solid surfaces are moved relative to one another (such as the rolling ball-on-disc technique). While the average shear can be estimated, the local shear in asperity–asperity contact could be many orders of magnitude higher. Any asperity–asperity contact can also result in wear/removal of the native oxide layer revealing the very reactive iron metal (Fe(0)). These contacts can also result in significant local heating/temperature rise which may lead to desorption or an apparent change in the activation energy of bonds breaking rather than the shear directly removing the adsorbed species. Therefore, the response of the system is convoluted by multiple coupled factors, while the true local shear remains ill-defined, making it challenging to determine the specific role of the shear.

In brief, there are a number of responses that may be expected for an adsorbed molecular layer under high shear. This may include desorption, structural changes (e.g., “tilting”), or molecule degradation. It is also expected that the strength of the adsorption of these layers will be important. For example, under very high shear a weakly bound physisorbed species is expected to desorb rather than break. In contrast a chemisorbed molecule with a stronger bond binding the molecule to the substrate, where the surface bond is stronger than the intra molecular bonding, might be expected to break rather than simply desorb.

In this present work, we aim to impose a well-defined shear field using fluid flow at the solid liquid interface (instead of solid–solid contact). We hope this will enable us to deconvolute and understand the effect of shear on the surface adsorbed layer from the other factors. A key challenge of the present work is to experimentally obtain high shear uniformly over a large area that is suitable for neutron reflectivity. A number of methods of obtaining uniformly high shear at the solid–liquid interface for neutron reflectivity studies include cone and plate rheometers^6^ (maximum shear, 10^2^ s^–1^), a tribometer^7^ (maximum shear, 10^3^ s^–1^), and a narrow gap flow cell^9^ (maximum shear, 10^4^ s^–1^). We have followed a narrow gap flow cell approach similar to that employed by Penfold et al.^9^

## Experimental Section

### Materials

Deuterium oxide (D_2_O) was obtained from Sigma (>99% purity, >99.9% D atom). Ultrapure water (UPW18.2 MΩ cm) was obtained using a Millipore water purification system. AOT (>99% purity), and palmitic acid (>99% purity) were obtained from Sigma.

The silicon single crystals (n-type material (111) face, 100 mm × 50 mm × 10 mm) for the NR measurements were purchased from Crystran and polished by the supplier to a very low surface roughness (quoted as ≤5Å roughness (RMS)). The crystals were cleaned in concentrated nitric acid for 3 h to remove organic contaminants, washed 20 times in ultrapure water, and allowed to soak overnight. The crystals were removed from the water and placed in a UV/ozone cleaner for 15 min to remove any remaining contaminants just before use.

The alumina single crystals (0001) were purchased from PiKem. These were cleaned with a mild piranha solution (5:4:1 H_2_O:H_2_SO_4_:H_2_O_2_) for 15 min at approximately 80 °C, washed 20 times in ultrapure water, and dried with N_2_, before a UV/ozone treatment.

### Shear Cell

A narrow gap flow cell, Figure 1, was used to impose shear at the solid–liquid interface. The flow cell includes a small gap (0.15 mm in our experiments) between two plates where the fluid is pumped through using a peristaltic pump. Although a peristaltic pump can give a pulsing flow, at the high fluid flow rates of interest here, this is expected to be minimal. The cell produces laminar Poiseuille flow adjacent to the solid surface of both the top and bottom plates. The maximum obtainable shear at the solid–liquid interface is 2 × 10^5^ s^–1^, with the mathematical description underlying the shear calculations presented in the Supporting Information. Similar shear rates have been previously achieved by Penfold et al.^9^ While in this paper “shear rate” is used to quantify the imposing shear, it is the shear stress that is important (connected by the fluid viscosity). The Supporting Information gives the details of the equations used to estimate the imposed shear field at the solid/liquid interface. The shear rate was measured through the volumetric flow rate through the cell. The pressure drop was measured using pressure sensors at both ends of the flow cell to ensure shear rates reported are reliable and not reduced due to the gap between the two plates expanding as a result of the high pressure. Flow behavior was additionally evaluated by introducing dye into the cell and visually confirming, by eye, that no stagnant regions or disrupted flow pathways were present.

**Figure 1:**
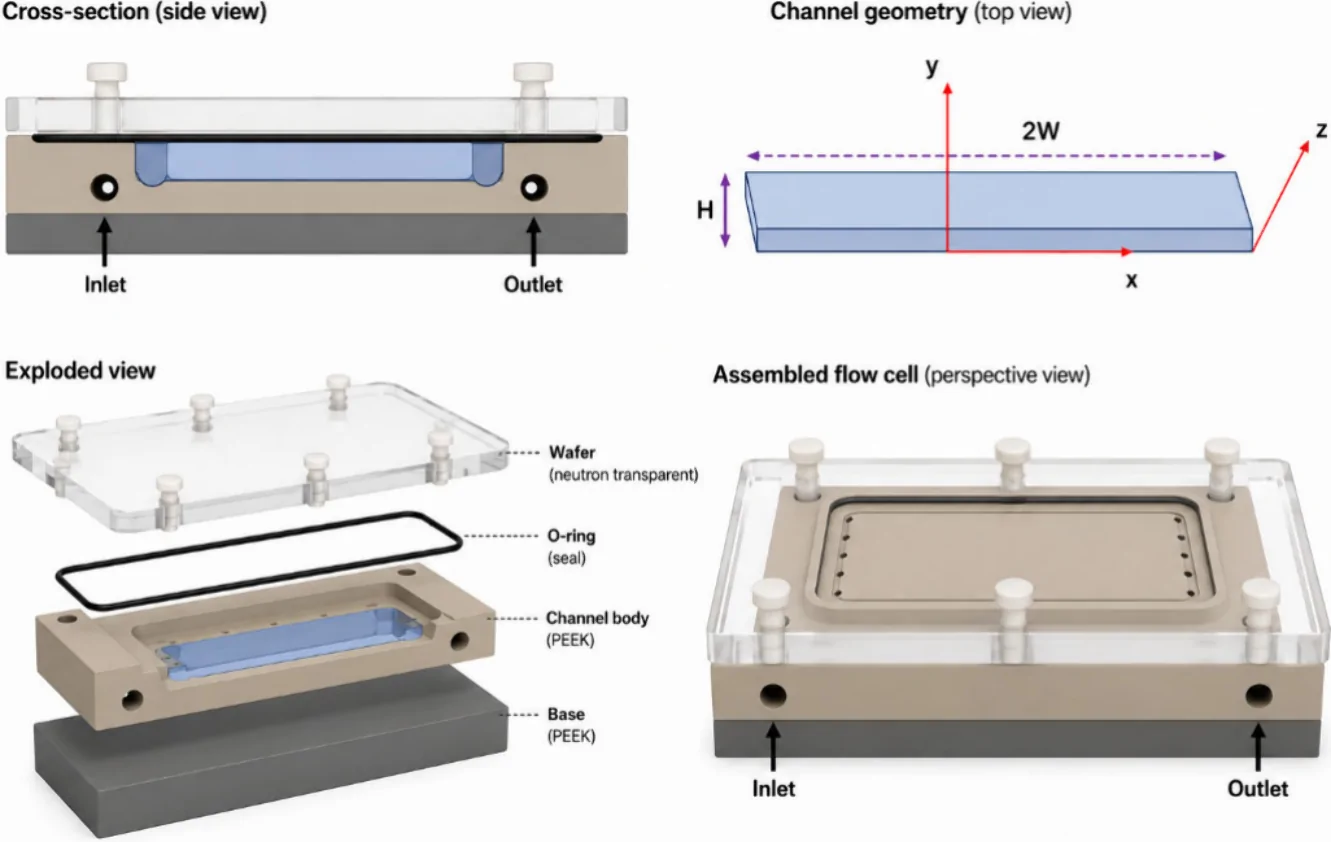
A schematic of the flow cell designed to obtain high shear at the solid–liquid interface. The fluid flows through a small gap, 0.15 mm between two plates. The inlet is split into 5 holes which splits the fluid flow from the inlet tube to ensure a uniform flow flux across the surface. The inlet and outlet are recessed to facilitate even, laminar flow across the cell width. A transparent glass is used here as the top plate for clarity. The cross-section of the flow area and the defined coordinate system used to model the fluid flow are also included. The *z*-axis is shifted for clarity.

### Molecular Layer Formation

*AOT:* The AOT-adsorbed films were formed by equilibration from a solution on to the alumina surface (as reported previously^6^). As described below, an AOT solution with a concentration of 2.5 mM, at the critical micelle concentration (CMC), was used where at equilibrium, an adsorbed bilayer is expected to form.^6^

*Palmitic Acid:* Palmitic acid is essentially insoluble in water and hence could not be adsorbed via solution. In this case, the material was deposited through spin coating onto silicon substrates. A palmitic acid solution (2.5 mg/mL) was formed by dissolving in chloroform which was then applied on the center of the stationary silicon substrate and subsequently rotated at high speed (4000 rpm) in order to spread the coating material by centrifugal force.^23^ Layer characterization is presented below.

**Neutron reflectometry** (NR) data were collected using the INTER instrument at the ISIS neutron facility, Rutherford Appleton Laboratory, U.K. Full details of the instrument may be found elsewhere.^24–26^ Specular reflection data were collected at incident angles of 0.5°, 1.0°, and 2.3°. Substrates were cleaned as specified above before being mounted in a custom-made aluminum cell with a PTFE trough on which the substrate, alumina or silicon, blocks were placed and clamped tightly to form a closed channel (as shown in Figure 1).

Collimation was used to control the beam footprint on the substrate to illuminate only the surface region within the trough and to maintain a constant d*q*/*q* resolution throughout the experiment. *q* is the momentum transfer vector (*z*-component) defined as

$$q=\frac{4\pi \sin\theta }{\lambda }$$

where θ and λ are the beam angle of incidence with respect to the surface plane and the wavelength, respectively. The cell was very slowly filled with the solution of interest, and a full set of data, measured at the 3 angles was collected and combined to give a single reflected intensity vs *q* plot for analysis (total measurement time was approximately 2 h). These measurements were then repeated under increasing shear rates. Fitting of the NR data was executed using GenX 2.0.0 software using the standard slab model approach.^27^

### Quartz Crystal Microbalance

A QCM with dissipation monitoring, Q-Sense E4 system, from Q-Sense, Sweden, was used for the in situ adsorption of AOT from aqueous solution onto alumina coated quartz sensors. Experiments were performed at the Nanoscience Centre, University of Cambridge. The Q-Sense E4 includes four sensors that can be used in a parallel configuration with temperature control. Full details of the technique and instrument can be found elsewhere.^28^ In brief, the principle behind this technique is that the resonant frequency of the sensor depends on the adsorbed mass. When surfactants adsorb onto the surface, the resonance frequency shifts (decreases with increasing mass), which is detected. The decrease in frequency (Δ*f*) can be related to the mass adsorbed (*m*) through the Sauerbrey equation:^29^

$$\Delta m=-\frac{C\Delta f}{n}$$

where *C* is a constant taken to be 17.7 ng cm^–2^ Hz^–1^ for the quartz crystals in this work, and *n* is the overtone number (1, 3, 5, ..., 13).

QCM sensors were thoroughly cleaned by sonicating in 99% ethanol (30 min), sonicated in ultrapure water (30 min), rinsed thoroughly with ultrapure water, dried with N_2_, and cleaned under UV-Ozone for 20 min. The QCM instrument was cleaned with flowing 99% ethanol (30 min) and rinsed thoroughly with ultrapure (30 min) before the sensors were loaded. Dissolved gases in surfactant solutions were removed by sonication. Ultrapure water was then flowed through (50 μL/min) for 2 h until the frequency of the sensors was stable. Afterward, the lowest surfactant concentration was pumped until a plateau value of the frequency was attained. The next (higher) surfactant concentration was then pumped onto the same crystal, without stopping the device or cleaning the crystal, until a new equilibrium was reached. This was repeated until the whole range of AOT concentrations of interest was completed. The crystal was then flushed with ultrapure water to investigate the reversibility of adsorption.

## Results and Discussion

### AOT Adsorption

To characterize the adsorption of AOT at the alumina–water interface, QCM was employed to measure the adsorption isotherm as a function of both concentration and temperature. These isotherms as a function of temperature enable the thermodynamic parameters such as enthalpy of adsorption to be determined. This enthalpy can be considered to be a measure of the binding strength of the surfactant to the substrate. Figure 2 presents experimental adsorption isotherms of AOT at the water–alumina interface. As expected, an increase in adsorbed amount with concentration is observed as well as a reduction in adsorbed amounts with increasing temperature (typical of exothermic adsorption). The corresponding Langmuir constant *K* at 25 °C for AOT adsorption is 3.3 mM^–1^, the enthalpy of adsorption is −37.8 kJ/mol, and the entropy change is −60 J/(mol K), determined from a Van’t Hoff plot (Figure 2).

**Figure 2:**
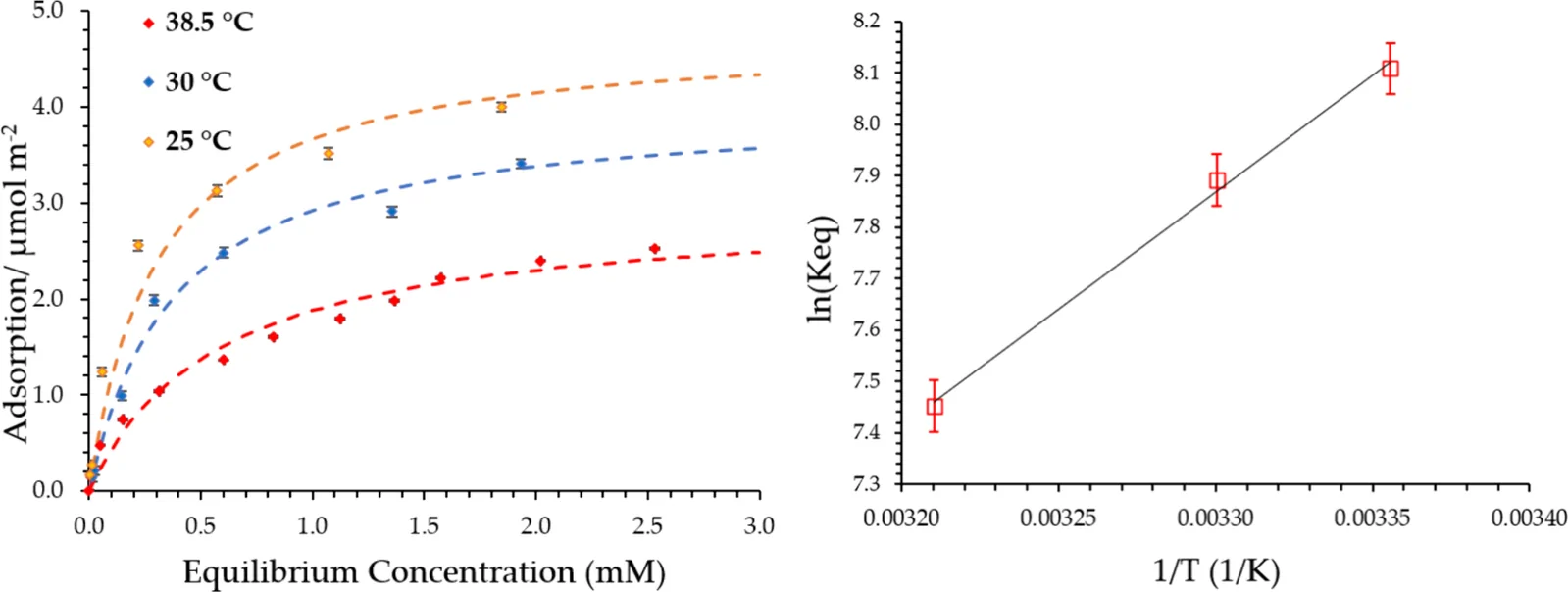
(Left) Adsorption isotherms of AOT from water onto alumina at different temperatures measured with QCM. The dashed lines are fitted Langmuir model to experimental data. (Right) Van’t Hoff plot of the equilibrium constant (*K*) as a function of inverse temperature.

At first sight, this adsorption enthalpy is much smaller than expected from formation of a chemical bond (typically several 100 kJ/mol) and, hence, is taken to indicate physisorption in this case. The isoelectric point of alumina is approximately at a pH of 8.^30,31^ Hence, at neutral pH, the surface is expected to be weakly cationic. The AOT has a negative charge, and hence we might expect a modest electrostatic interaction. However, it is important to note that the adsorption is essentially measuring an exchange reaction where initially adsorbed water molecules are replaced by the AOT. Hence, the enthalpy we measure is expected to be less than the binding of the AOT to the sapphire, the amount it is lower by is the binding energy of the water molecules, and there may be several bound water molecules replaced by the adsorbing AOT. There may also be released water molecules that were solvating the AOT headgroup on adsorption. Hence, in summary, although the measured enthalpy indicates a modest physisorption, the actual strength of AOT–surface may be significantly more than this.

### AOT System Static Conditions (NR)

Initially, AOT on the surface of alumina from D_2_O was characterized at the CMC (2.5 mM) under static flow conditions, where a single bilayer is expected to adsorb at the solid–liquid interface.^6,30^ A large Kiessig fringe was observed in the reflectivity profile (Figure 3) corresponding to an AOT adsorbed layer at the interface. These data are very different from those of the bare alumina substrate, clearly indicating significant adsorption, in agreement with previous studies.^6,30^

**Figure 3:**
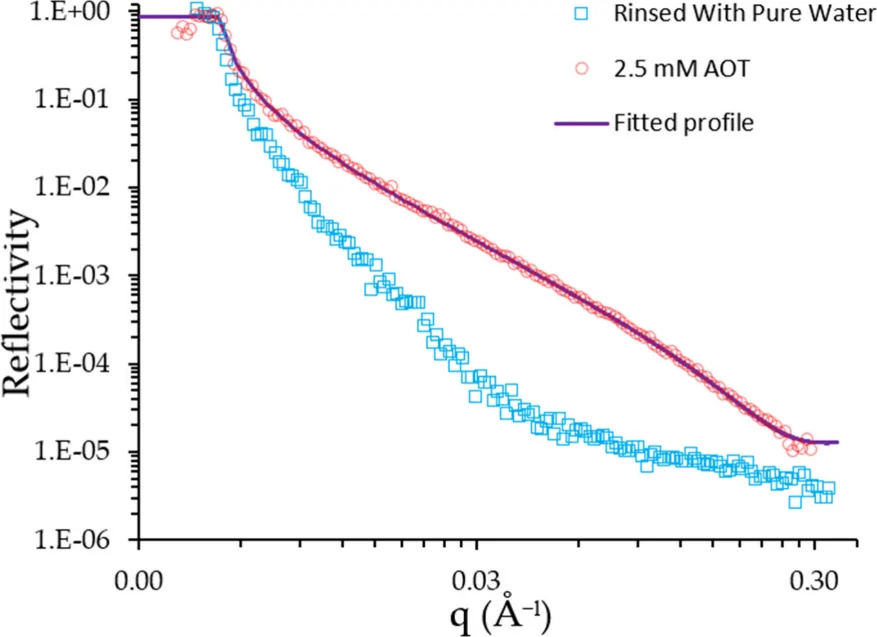
Reflectivity profile and the corresponding fitted profile of the AOT bilayer adsorbed at the D_2_O–alumina interface under static conditions. The system was subsequently rinsed thoroughly with clean water before measuring the bare surface profile. While the large Kiessig fringe is removed suggesting most of the AOT bilayer is removed from the surface, it is likely that a small amount of AOT remains.

Previous studies performed by Hellsing et al.^30^ reported the AOT bilayer structure which consists of a symmetric head–tail–tail–head adsorbed at the alumina–water interface. Therefore, a similar 3-layer approach was used to model a bilayer with a symmetric head–tail–head model. The thickness and scattering length density (SLD) of each headgroup region were constrained to be the same. The fitted parameters are presented in Table 1. The resultant fitted SLDs can be used to calculate the structure parameters of the bilayer, which include area per molecule. The obtained parameters are in good agreement with Hellsing et al’s study which corresponds to an area per molecule of 57 Å^2^ and 12 water molecules per headgroup, with a total thickness of 37 Å. A slightly lower SLD for D_2_O was obtained from the fit; this is attributed to a very small amount of exchange of D_2_O with H_2_O from the air or a small amount of H_2_O remaining in the cell on rinsing with D_2_O after H_2_O. These results suggest that the previously reported adsorbed AOT bilayer was successfully reproduced in this experiment.

**Table 1:** Fitted Parameters, Consisting of a Symmetrical AOT Bilayer at the Alumina–D_2_O Interface Obtained from the Bare Surface NR Profiles^a^

	fitted parameter		
medium	thickness (Å)	SLD × 10^–6^ (Å^–2^)	roughness (Å)
Al_2_O_3_	∞	5.75^a^	3 ± 2
AOT head	9.1 ± 1.5	6.12	2 ± 1
AOT tail	18.9 ± 2	–0.14^a^	2 ± 1
AOT head	9.1 ± 1.5	6.12	7 ± 3
D_2_O solution	∞	6.2 ± 0.4	

a Values constrained to those reported in the literature.^30^

### AOT under Shear

The AOT adsorbed layer was subjected to a number of increasing shear rates and the reflectivity profile was measured. Figure 4 shows the reflectivity from the lowest and highest shear rates obtainable (3 × 10^5^ s^–1^), alongside the static and subsequently rinsed measurements, with no variation in the reflectivity profile between the highest and lowest shear rates. It is important to note that the bulk solution used to shear the surface was also at the 2.5 mM AOT solution concentration to avoid changes in the surface adsorbed amount and stability to due to changes in bulk concentration. This result indicates that even at this very high shear there is no significant impact on the adsorbed layer (for clarity only the reflectivity profiles at the lowest and the highest shear rates are shown). A small change is seen between the measurements under shear compared to the static measurement, with a slight increase in the fringe intensity and a sloping of the low-*q* region (critical edge). This sloping is unexpected but self-consistent and could be from the introduction of some small bubbles into the cell (which were unavoidable owing to the large tubing used to obtain the higher shear rates). This effect dominates at lower *q* values and does not affect the conclusion that the adsorbed AOT bilayer remained on the surface under these shear rates. The increase in fringe intensity could indicate a small change in bilayer thickness, a reduction in D_2_O in the adsorbed layer or link to the air bubbles. With only a single contrast measured it is not possible to separate these options.

**Figure 4:**
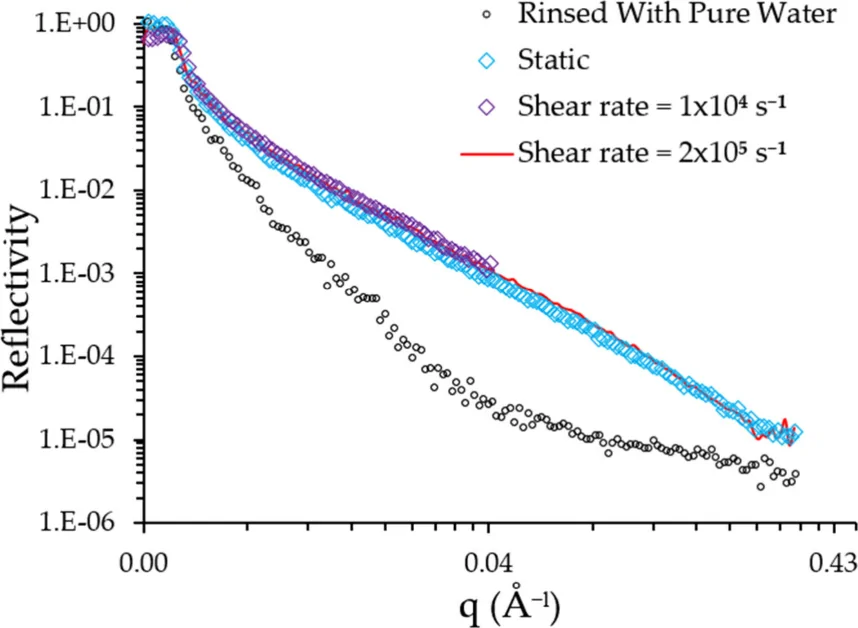
Reflectivity profile of the AOT bilayer under static, sheared conditions and the bare alumina surface (after thorough rinsing with pure water). Note the reflectivity profile at shear rate of 2 × 10^5^ s^–1^ is plotted as a line for visual clarity and is not a model fitting.

Figure 4 also shows the NR data when the sample has been thoroughly rinsed with pure water. These data demonstrate a very big difference in reflectivity profile. The large Kiessig fringe is no longer present in the reflectivity profile, indicating that the bilayer is removed. Importantly, this indicates that although not removed or changed by the highest level of applied shear imposed, the layer is removable by rinsing the system with pure water (i.e., dissolving the layer). Therefore, even the maximum shear rate obtainable in this experiment is insufficient to perturb the adsorbed bilayer.

It is important to note that since the bulk solution is at the cmc, there is a chemical potential favoring readsorption. However, what is seen in the neutron reflection experiment will depend on how much the shear removes and how fast, relative to the speed at which it can be replaced from solution. Hence, all we can conclude here is that either the shear has very little effect on the layer or the rate of readsorption of any layer removal is very fast.

### Palmitic Acid Adsorption

As palmitic acid is essentially insoluble in water, it is not possible to measure the adsorption isotherm on the silicon substrate in water. Alternatively, the adsorption of palmitic acid onto the silicon surface from the nonaqueous solvent, cyclohexane, was measured using neutron reflectivity (Figure 5). Due to the significant role solvents play in surface adsorption, this is not directly comparable with palmitic acid adsorption from aqueous solvent; however, it still provides important insights into the surface binding strength of palmitic acid to the silicon surface. The data from the bare silicon surface in pure deuterated cyclohexane and as well as a 35 mM of palmitic acid in cyclohexane solution show no significant change in the reflectivity profile. Hence, we conclude that there is no significant adsorption of the palmitic acid onto the silicon surface from cyclohexane. Given this lack of preferential adsorption of fatty acids onto the silicon surface compared to remaining in the bulk solution, it suggests that there is minimal palmitic acid–silicon surface interaction. Therefore, for a palmitic acid layer that is spin-coated onto the surface, the interaction and binding strength of the palmitic acid to the surface are expected to be very weak. While it is possible to measure adsorption isotherm using silica powder (nonaqueous solvent), in related work, we have shown that silica surface is not a good representative of silicon surfaces.^32^ The adsorption energy of fatty acids on silica powder is reported at around 20 kJ/mol,^33^ which is much lower than measured in the current study for AOT on alumina. Indeed, (hydroxylated) alumina has a much higher surface free energy than (hydroxylated) silica and thus promotes stronger adsorption with the surfactants. This supports our hypothesis that AOT is harder to remove from alumina with shear than palmitic acid on silica (even easier from silicon surface) due to stronger adsorption.

**Figure 5:**
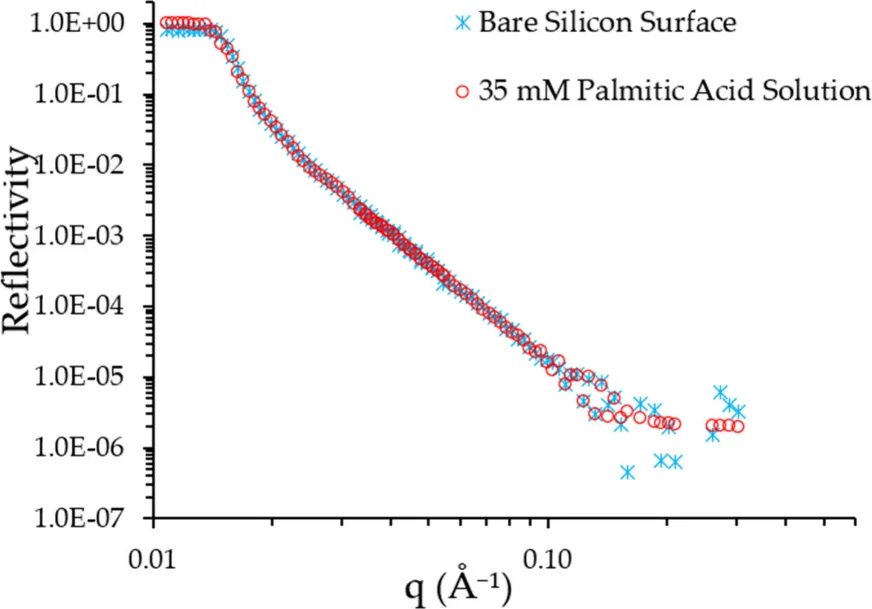
Neutron reflectivity profile from silicon surface exposed to pure cyclohexane and solutions of palmitic acid.

It is very important to note that adsorption thermodynamics and the layer structure are expected to be strongly solvent dependent. This is a very simplified assessment of the molecule’s affinity for the surface. It is expected that water as a solvent will, at least partially, solvate the hydrophilic SiOH groups on the surface (the silicon will be covered by the native oxide) and associate with the fatty acid head groups.^34–36^ These water molecules will need to be removed for the acid to bind to the silicon, which is expected to be unfavorable and endothermic. Hence, both these effects will further disfavor adsorption on to the silicon. In contrast the cyclohexane is a much better solvent for the palmitic acid than water and this may be the origin of the lack of adsorption equilibrated from a solution in cyclohexane.

### Palmitic Acid Multilayer System (NR)

Palmitic acid was spin-coated onto large silicon blocks (10 cm by 5 cm) as outlined above, with some visible inhomogeneity. The block was subsequently placed in the shear cell very carefully to prevent delamination of the multilayer. The reflectivity profile of the multilayer was measured twice 24 h apart under static conditions and showed no changes. Hence, it can be concluded that the surface layer is stable on the time scale of the experiment.

Within the measured *Q*-range, a Bragg peak is present at a *Q*-value of 0.1735 Å^–1^ which indicates a repeating layered arrangement of the palmitic acid with a layer thickness of 36 Å. The length of a molecule of palmitic acid is in the region of 15–18 Å which suggests a deposited stack of palmitic acid bilayers.^37^ This head-to-head orientation is typical of long chain fatty acids in the bulk crystals.

In the static case, air bubbles were present in the shear cell, and at low shear, further bubble entrainment was observed. Under these conditions, the reflectivity exhibited significant off-specular scattering and reduced intensity below the critical edge (Figure 6). While air bubbles may contribute to this behavior, the reduced critical-edge intensity is also consistent with, and likely dominated by, Yoneda scattering from in-plane correlated roughness of the thick PA layer, as seen in the detector images. Given the challenges with sample preparation, another contributing factor to the off-specular scattering could be micrometer-scaled holes in the multilayer stack due to incomplete coverage after spin-coating. The evolution of this feature under shear and its improvement at higher shear rates as the Bragg peak disappears are consistent with this interpretation. Some remaining rounding of the critical edge at the highest shear rates may still indicate the residual effect of bubbles. These factors make quantitative fitting of the specular reflectivity unreliable, and so the analysis focuses on qualitative changes in the Bragg peak rather than absolute structural parameters of the adsorbed layer.

**Figure 6:**
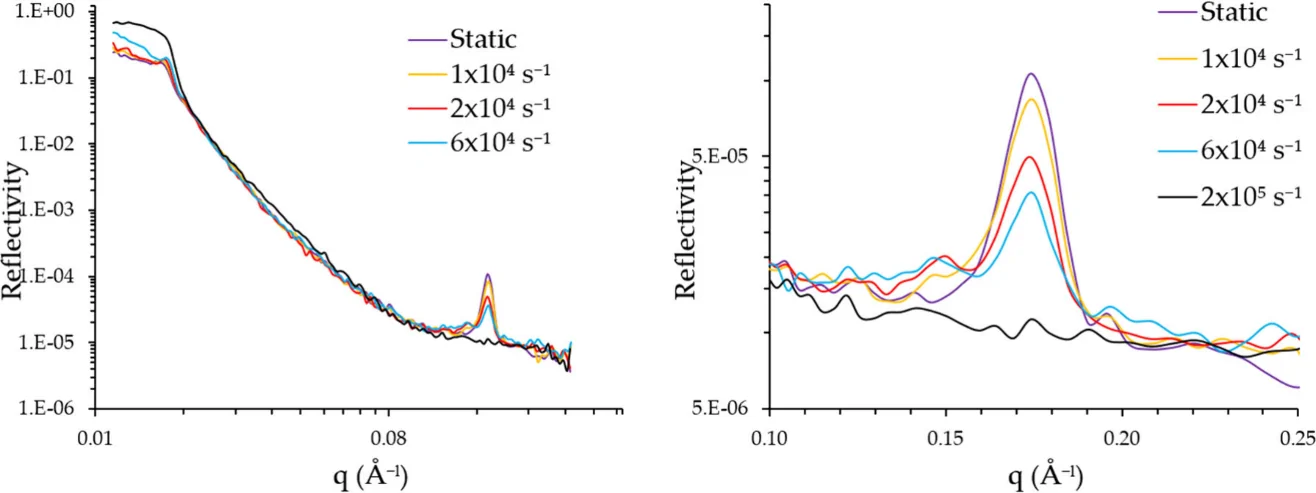
Reflectivity profile of the palmitic acid multilayer as a function of shear showing a reduction in Bragg intensity with shear. The experiments at each shear rate were measured for approximately 50 min, as the Bragg features change with time. The reported reflectivity is an average reflectivity profile over the 50 min measurements.

The effect of shear on reflectivity from this deposited multilayer is shown in Figure 6. The sharp peak at 0.17 Å^–1^ from the palmitic acid, lamellae which is initially very intense, becomes weaker and broader with increasing shear (but remains at the same *Q* value). Importantly at the highest shear rate the feature disappears. This is similar to the AOT multilayer system studied by Welbourn et al.;^6^ however, in that case a surface bilayer remained. A drop in peak intensity and broadening of the Bragg peaks were found, alongside a loss in the off-specular (Yoneda) scattering around the critical edge (i.e., decreased correlated film roughness). This suggests a possible “de-lamination” of the lamella sheets. The reflectivity curve from the highest shear rate is consistent with complete removal of the palmitic acid.

Although quantitative analysis of the Bragg peak is not feasible because of the complications with the sample, qualitative changes in the peak still provide important insights. Parameters from a simplified Lorentzian fit to the Bragg peak with shear conditions are shown in Figure 7. This shows a steady loss in peak height with shear rate with a corresponding increase in peak width, while the peak center remained constant. This is consistent with the proposed delamination, while the makeup of remaining layers remained approximately constant.

**Figure 7:**
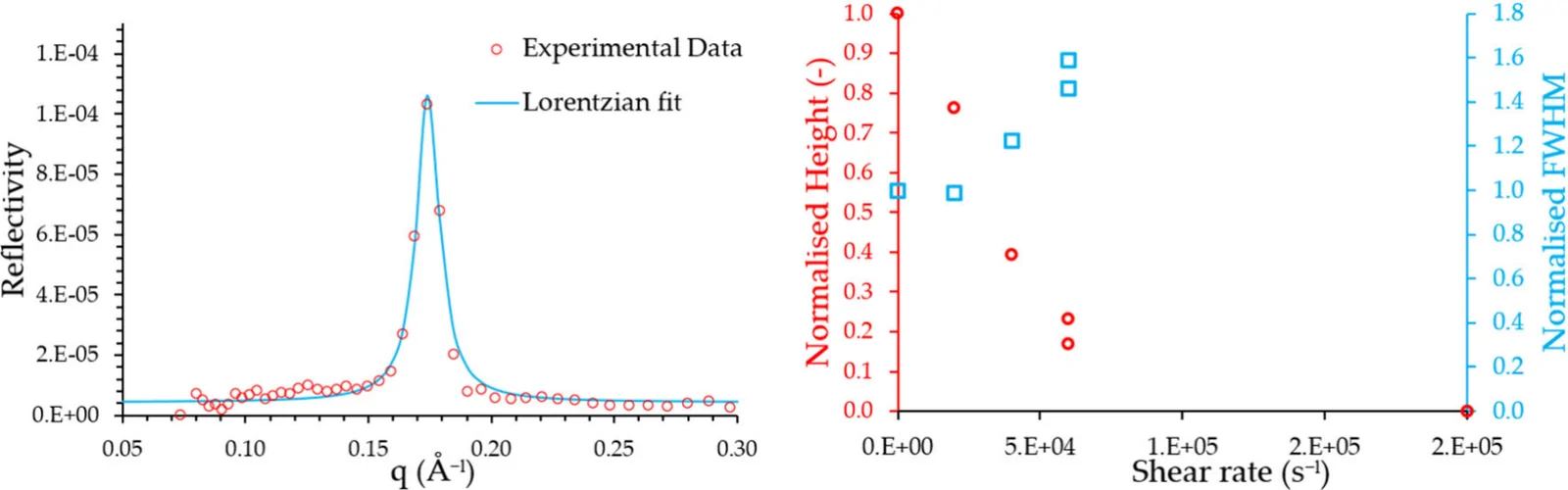
(Left) An example of the Bragg fitting procedure; a Lorentzian profile fitted to the static data. (Right) Normalized peak height (red) and fwhm (blue) as a function of shear. The plotted values are intended to demonstrate qualitative variation only, and the exact numerical values should not be overinterpreted.

### Surface Adsorbed Bilayer

While it is evident that the initially deposited multilayer is delaminated/removed by the highest shear rate that can be imposed, it is important to consider whether the surface-bound bilayer (or monolayer) remains. At this high flow rate, where the bubbles have been removed, the NR profiles can be fitted. The reflectivity profile shown in Figure 8 was fit to a clean silicon surface, using parameters from the literature (Table 2). This fit captures the reflectivity profile rather well, suggesting that even the surface bilayer is removed at the high shear.

**Table 2:** Fitted Parameters for Bare Silicon Surface in D_2_O Solution^a^

	fitted parameter		
medium	thickness (Å)	SLD × 10^–6^ (Å^–2^)	roughness (Å)
Si	∞	2.07^a^	9 ± 3
SiO_2_	23 ± 3	3.41^a^	3 ± 2
D_2_O	∞	6.1 ± 0.6	

a Values are fixed to expected values from the literature.

**Figure 8:**
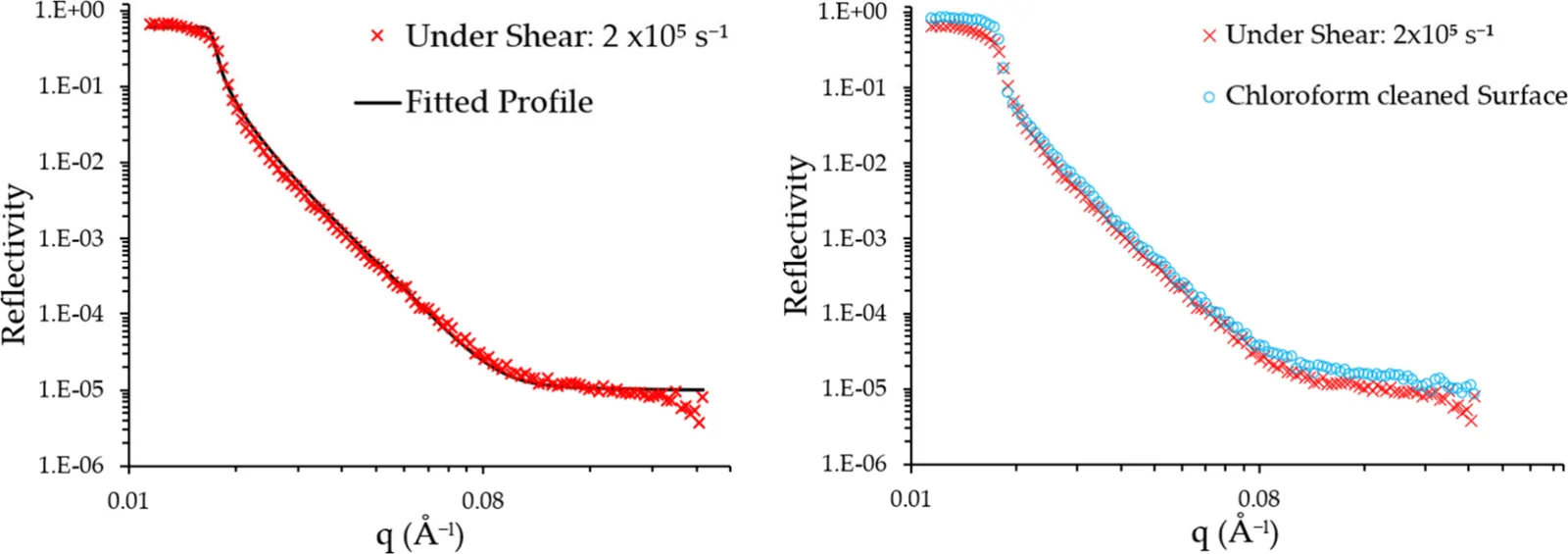
(Left) The reflectivity profile of palmitic acid sample at the highest shear and the corresponding fit assuming a clean silicon surface. (Right) Reflectivity of the sample after being cleaned thoroughly with chloroform.

Furthermore, the silicon wafer was repeatedly rinsed with chloroform (a good solvent for the fatty acid) to remove any remaining palmitic acid layers. The reflectivity profile at 2 × 10^5^ s^–1^ compared to the chloroform rinsed wafer is shown in Figure 8 (right). There is no significant change in the reflectivity profile nor are there any Kiessig fringes, which further supports the conclusion that no surface bilayer remains.

This result contrasts with the AOT system (where the bilayer adjacent to the solid surface is not removed) even at the highest shear rates. As discussed above, the palmitic acid is essentially insoluble in water. Therefore, the layers are not “dissolved” from the surface but apparently “rubbed” off by the fluid flow, i.e., via a “mechanical” effect.

### Discussion. Adsorption Strength: “Strong” and “Weak” Layers

It is important to note that AOT is soluble in water and preferentially adsorbs onto the surface from bulk solution in a dynamic equilibrium, any delamination of the surface-bound bilayer or multilayer under shear would be replenished if the shear is removed (with the appropriate time scale). Conversely, PA is insoluble in water; therefore, there is no dynamic equilibrium of adsorption and desorption here. Any removal/delamination of the PA multilayer will not be replenished. The effect of shear on this multilayer is purely mechanical, and the effect of shear in both systems is expected to be rather different.

#### The Bilayer Adjacent to the Solid Surfaces

The different effects of shear on these adsorbed layers may be attributed to the relative strength of binding of the molecules to the solid surfaces. Any energy of adsorption must be overcome as an activation barrier, or the molecule remains at the surface. Therefore, a minimum shear force may be required to remove a molecule bound on the surface. Hence, a strongly bound species (e.g., AOT/alumina) will require a far higher shear rate than a weakly bound species (palmitic acid/silicon) to be removed. This activation energy must be at least as big as the energy of adsorption, which therefore represents a lower bound. Hence the enthalpy (heat) of adsorption and the related parameter *K* (Langmuir isotherm constant) in some sense reflect the additive’s affinity for the surface/“strength” of binding and hence the barrier to removal by the shear.

The adsorption of fatty acids on silicon surfaces from the nonaqueous solvent cyclohexane presented above suggests that palmitic acid does not adsorb at all and hence does not have any affinity for the surface (subject to the very significant caveats above). In contrast, the adsorption studies have demonstrated that the AOT bilayer adsorbs more strongly onto the sapphire surface than palmitic acid binds to the silicon surface. This large difference in binding strength to the surface may be the reason why the surface PA bilayer was removed with shear, but the AOT surface bilayer was not perturbed even at the highest shear rate possible.

#### Multilayer Stacks of Bilayers

Previous reported studies^6^ demonstrate that the AOT multilayer stack of bilayers is very weak and removable at shear rates as low as 0.5 s^–1^ (even though the bilayer on the surface is much more robust, to even the highest shear rates). In this study high shear rates can delaminate the PA multilayer stack (Figure 8).

When considering the strength of a multilayer, there are two parts: the strength of binding of the first layer/bilayer to the surface and the strength of binding between the upper layers. Commonly, the strength of the binding between these upper layers may be assumed to be the same throughout the multilayer system. From the common BET equation with affinity constants, *K*_L_ for the first monolayer/bilayer and *K*_s_ for the ensuing multilayers, *K*_s_ can be linked to the solubility limit, *C*_s_, as *K*_s_ = 1/*C*_s_.^38–40^ The solubility of AOT in water^41^ (32 mM) is significantly higher than that of palmitic acid in water^42^ (0.028 mM); therefore, we can infer that the strength of multilayers in palmitic acid is higher than that of the AOT. This might explain why the multilayer stacks are more easily removed by shear for AOT than for palmitic acid.

Figure 9 gives a schematic of the structure of AOT (top) and PA (bottom) multilayers in water. The AOT bilayer thickness (35–37 Å) is approximately the same as that of a palmitic acid bilayer, however, the separation gap between the layers is significantly different in the two systems. There is a gap (filled with water molecules) of 148 Å between AOT bilayers. These water molecules are expected to easily move/slide which suggests that the multilayer is weak and can be perturbed with relatively low shear.

**Figure 9:**
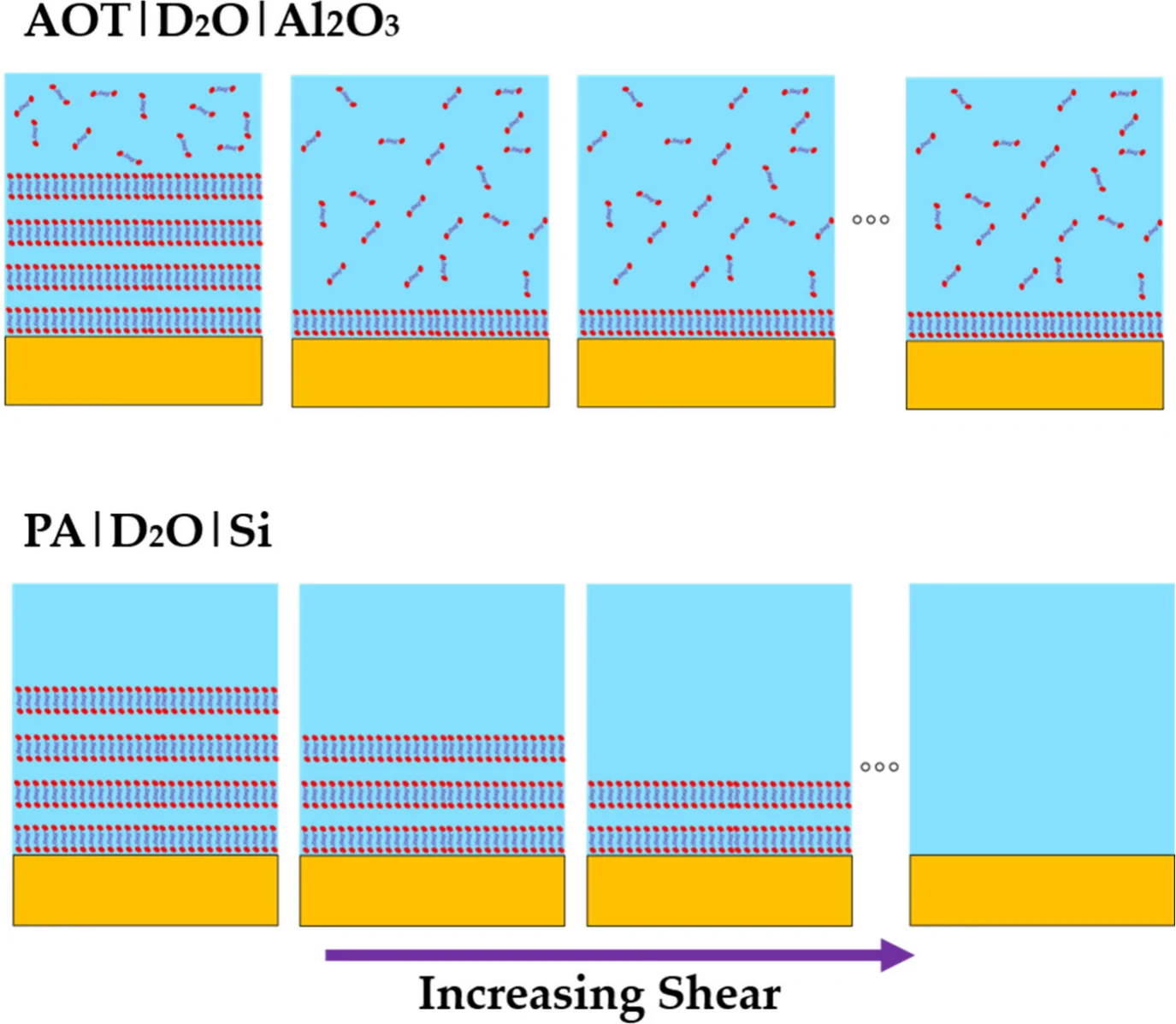
A schematic of the structure of AOT (top) and PA (bottom) multilayers in water. The AOT layer preferentially adsorbs from solution, whereas the insoluble palmitic acid layer is spin coated onto the surface. The schematic demonstrates the systems response to increased levels of shear with AOT multilayer removed at low shear, but the surface bilayer persists even at the highest shear rate possible. The removal of the palmitic acid multilayer is more gradual with increasing levels of shear; however, even the surface bilayer is removed at the highest shear rate possible.

In contrast, the gap between the PA bilayers is very small (they are essentially in contact). The acid head groups are possibly bound to the next bilayer by strong hydrogen bonds, i.e., self-association.^34–36^ However, there are expected to be weaker alkyl chain to alkyl chain interactions that may facilitate slippage/shear. However, these palmitic–palmitic interactions are again expected to be higher than those of AOT interbilayer interactions.

Hence, we conclude that the relatively strong palmitic–palmitic interactions lead to breakdown of the bilayer–multilayer stack at a higher shear rate than for AOT multilayer. However, the stronger specific interactions of the AOT with alumina are relatively stronger than palmitic acid and silicon so that high shear cannot remove the AOT bilayer on the alumina but can remove the palmitic acid bilayer on the silicon.

## Conclusions

This study demonstrates the use of in situ neutron reflectometry with a narrow-gap flow cell to directly probe the response of molecular layers at buried solid–liquid interfaces under well-defined, high-shear conditions. Shear rates of up to 2 × 10^5^ s^–1^ were applied to two contrasting systems: an AOT bilayer adsorbed from aqueous solution onto alumina and a spin-coated palmitic acid multilayer on silicon. The results demonstrate that the response of these molecular layers cannot be predicted from the magnitude of the applied shear alone but instead depends on a balance between the imposed shear stress and binding affinity to the surface.

The surface AOT bilayer remained essentially unchanged, even at the highest shear imposed. This stability is particularly notable because the layer could subsequently be removed by rinsing with pure water, i.e., “dissolving” the layer. The result therefore suggests that resistance to mechanical perturbation under shear is distinct from thermodynamic stability following a change in bulk composition. Independent adsorption measurements demonstrate a stronger affinity of AOT for alumina surface than the palmitic acid on silicon surface. Consequently, these results demonstrate that either the adsorbed bilayer is intrinsically stable under the applied shear or that any removal occurs more slowly than replenishment from solution.

In contrast, the palmitic acid multilayer progressively lost structural order with increasing shear. At the highest shear rate imposed, the reflectivity profile was consistent with the removal of not only the outer multilayer stack but also the layer directly adjacent to the silicon surface. The lack of adsorption of palmitic acid from cyclohexane onto silicon suggests that palmitic acid has a comparatively weak affinity for this substrate. At the same time, the gradual disruption of the multilayer indicates stronger palmitic acid–palmitic acid cohesion within the deposited film. These results distinguish the strength of binding between adjacent molecular layers from the strength of binding between the first layer and the substrate surface: strong interlayer association can delay multilayer delamination, but it does not necessarily ensure retention of the surface layer when the surface-bound layer itself is weakly anchored.

## Supplementary Material



## Notes

Experiment measurements and mathematical description of the flow profile inside the cell; experimental measurements include pressure measurements across the cell to validate the mathematical model used to calculate the shear rates reported in the paper (PDF)
