# Supplementary material for: In Situ High Shear Perturbation of Surface Molecular Layers with Neutron Reflectivity
Source: Langmuir. 2026 Aug 10;42(33):24543–52. doi: 10.1021/acs.langmuir.6c03616 (PMC13523748; doi:10.1021/acs.langmuir.6c03616)
Supplement: Supplementary file 1 [file la6c03616_si_001.pdf]

Supporting Information

In-situ High Shear Perturbation of Surface Molecular Layers with Neutron Reflectivity

Najib Sharifi[1*], Rebecca J.L. Welbourn[2,3], Stuart M. Clarke[1]
[1]Institute for Energy and Environmental Flows and Yusuf Hamied Department of Chemistry, University of Cambridge, Cambridge, CB2 1EW, UK.
[2]ISIS Neutron & Muon Source, Rutherford Appleton Laboratory, STFC, Chilton, Didcot, Oxon, OX11 0QX, UK
[3]Neutron Scattering Division, ORNL, Oak Ridge, Tennessee 37831, United States

*Address correspondence to this author: najib.sharifi@rdm.ox.ac.uk

Calculation of the surface shear conditions

The velocity profile across a cross-sectional area of the flow cell can be calculated through the Navier-Stokes (NS) equation[1]. At steady-state, the velocity profile, U, of a fully developed Poiseuille flow is given by:

$$U(y,x) = \frac{2H^2}{\pi^3\mu}\left(-\frac{dP}{dz}\right)\sum_{n=1}^{\infty}\left(\frac{1-(-1)^n}{n^3}\right)\left(1-\frac{\cosh\left(\frac{n\pi x}{H}\right)}{\cosh\left(\frac{n\pi w}{H}\right)}\right)\sin\left(\frac{n\pi y}{H}\right)$$

Where H is the gap between the parallel plates, μ is the fluid viscosity, P is the pressure and x,y,z are the cartesian coordinates of the narrow gap as schematically demonstrated in Figure 1. This equation is in line with reported well-established solutions in the literature[1]. The corresponding shear rate at the solid-liquid interface is given by:

$$\gamma = \frac{\partial U}{\partial y}(x,0) = \frac{2H}{\pi^2\mu}\left(-\frac{dP}{dz}\right)\sum_{n=1}^{\infty}\left(\frac{1-(-1)^n}{n^2}\right)\left(1-\frac{\cosh\left(\frac{n\pi x}{H}\right)}{\cosh\left(\frac{n\pi w}{H}\right)}\right)\sin\left(\frac{n\pi y}{H}\right)$$

The maximum achievable flowrate in our system is 3.7×10^{-5} m^3/s, with a corresponding shear rate of 3×10^{5} s^{-1} at the solid-liquid interface.

The flowrate was measured by measuring the amount of fluid pumped over 5 minutes (Figure S1). The pressure drops across the cell was measured using pressure sensors at the inlet and outlet of the pressure cell. Interestingly above 300 RPM, the pressure drop measured across the cell rises significantly more than the predicted value. This is attributed to the losses at the inlet and outlet of the cell where the flow changes direction twice, each time moving perpendicular to the previous flow direction (Figure 1) this is likely to result in a relatively large pressure drop which becomes more significant at higher flowrates.

Given the relatively high pressures inside the narrow channel, the key concern is whether this high pressure can result in material deformation or the gap between the two plates increasing which would result in a decrease in the shear stress induced at the solid-liquid interface; this is undesirable. Any widening of the gap would result in a decrease in pressure drop which the results suggest does not happen, giving more confidence in the induced shear rate presented in this paper.

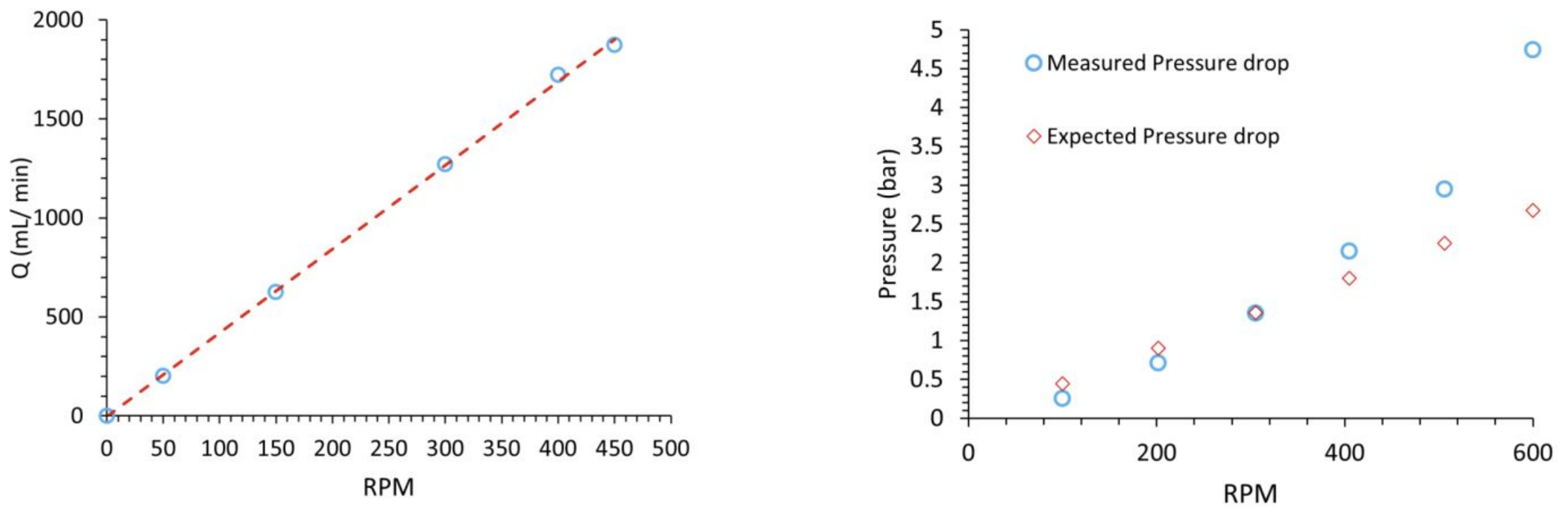

Figure S1. L) Flowrate measured as a function of the pump RPM showing a linear relationship as expected. R) The theoretical and experimentally measured pressure drop across the cell as a function of pump RPM.

References

1. Acheson, D. J. Elementary Fluid Dynamics, Oxford Applied Mathematics and Computing Science Series, *Oxford University Press*, 1990, ISBN 978-0-19-859679-0
